# “Such a different type of tiredness”: people with brain tumour, their caregivers’, and healthcare professionals’ qualitative perceptions of cancer-related fatigue

**DOI:** 10.1007/s11764-024-01691-3

**Published:** 2024-10-15

**Authors:** R. Campbell, J. M. Shaw, T. Carlick, H. Banks, M. M. Faris, M. S. Jeon, D. M. Legge, C. Foster, R. Leonard, R. J. Chan, M. R. Agar, A. Miller, H. M. Dhillon

**Affiliations:** 1 Psycho-Oncology Cooperative Research Group, School of Psychology, Faculty of Science, The University of Sydney, Camperdown, Sydney, NSW Australia; 2 Centre for Medical Psychology and Evidence-Based Decision-Making (CeMPED), School of Psychology, Faculty of Science, The University of Sydney, Sydney, NSW Australia; 3 Oliva Newton-John Cancer and Wellness Centre, Austin Hospital, Heidelberg, Melbourne, Australia; 4 Faculty of Health Sciences, Curtin School of Nursing/Curtin Health Innovation Research Institute, Curtin University, Bentley, Perth, Australia; 5 Centre for Psychosocial Research in Cancer (CentRIC), School of Health Sciences, University of Southampton, Southampton, UK; 6 Brain Cancer Collective, Trials Centre, NHMRC Clinical, University of Sydney, Sydney, NSW Australia; 7 Caring Futures Institute, College of Nursing and Health Sciences, Flinders University, Bedford Park, Adelaide, SA Australia; 8 Improving Palliative, Aged and Chronic Care Through Clinical Research and Translation (IMPACCT), Faculty of Health, University of Technology Sydney, Broadway, Sydney, NSW Australia; 9 Community Advisory Group, Psycho-Oncology Cooperative Research Group, The University of Sydney, Sydney, NSW Australia

**Keywords:** Cancer-related fatigue, Brain cancer, Primary brain tumour, Caregiver, Healthcare professional, Qualitative research

## Abstract

**Purpose:**

Cancer-related fatigue (CRF) is one of the most common symptoms reported by people with primary brain tumour (BT). Previous research predominantly examined CRF using quantitative assessments, failing to capture the rich insight garnered from exploring individuals’ lived experiences. We addressed this gap by qualitatively exploring people with BTs’ experiences of CRF.

**Methods:**

Semi-structured interviews were conducted with people with BT, their caregivers, and healthcare professionals (HCPs) who care for them. Interviews explored the experience, impact, and management of CRF, including types of support provided by HCPs. Data were analysed using reflexive thematic analysis.

**Results:**

Forty participants were interviewed (24 people with BT, 5 caregivers, 11 HCPs). Qualitative analysis identified four themes: pervasiveness of CRF; impacts of CRF; advice and support; and self-management strategies. CRF was described as an almost universal symptom with physical, emotional, and cognitive aspects and profound psychosocial and functional impacts. HCPs reported assessing fatigue and providing management support. Yet, people with BT and caregivers reported CRF assessment and support were rarely received. Consequently, people with BT developed their own management strategies. All participants identified a lack of CRF information resources and interventions specific to people with BT.

**Conclusion:**

Our findings provide rich insight into the pervasive, debilitating impact of CRF in people with BT and highlight the lack of BT-specific CRF support and information available.

**Implications for cancer survivors:**

There is a critical need for evidence-based fatigue interventions and information resources tailored to the needs of people with BT.

**Supplementary Information:**

The online version contains supplementary material available at 10.1007/s11764-024-01691-3.

## Introduction

Primary brain tumours are a heterogenous group of tumours arising within the central nervous system [1]. Gliomas are the most common malignant primary brain tumour with overall 5-year survival of 22% [2]. Treatment for malignant brain tumours typically consists of surgery, chemotherapy, and radiation therapy [3]. Due to tumour location and localised treatments, people living with brain tumour (BT) experience a multitude of burdensome symptoms, behavioural changes, and functional deficits, adversely affecting their quality of life [4–6].

Cancer-related fatigue (CRF) is one of the most prevalent and distressing symptoms reported by people with BT throughout the disease and treatment trajectory [5, 7–9]. CRF is commonly defined as “a distressing, persistent, subjective sense of physical, emotional, and/or cognitive tiredness or exhaustion related to cancer or cancer treatment, that is not proportional to recent activity and interferes with usual functioning” [10]. The prevalence of CRF among people with low grade gliomas is between 39 and 77% [11], with approximately 40% reporting severe CRF up to 8 years after completing treatment [12]. Of those with high grade gliomas, 48% report CRF after initial surgery [8], and up to 94% report CRF following tumour recurrence [5]. Increasing evidence indicates CRF is an independent prognostic factor for overall survival in people with BT [7, 13] and can have a profound adverse impact on daily life [14], underscoring the critical need for research to address CRF in this population.

Previous studies examining CRF in people with BT predominantly used quantitative self-report measures to assess fatigue [5, 7–9, 11–13]. The majority used either single- or multi-item unidimensional scales to assess fatigue severity [7–9] or physical aspects of fatigue [5, 13], providing limited insight into the subjective experience of fatigue. Previous qualitative research mainly examined adjustment to living with BT and managing overall symptom burden, also yielding little insight into the experience and impact of individual symptoms such as CRF [e.g.[15–17]]. Moreover, very few interventions targeting CRF have been evaluated in people with BT [18]. Thus, rich insight is needed into the experience of CRF, its effect on everyday life, and how it is managed to identify gaps in practice and inform optimal supportive care for this population. We aimed to use qualitative methodology to garner in-depth understanding of the experiences and impacts of CRF among people with BT and explore management strategies.

## Methods

### Study design

This qualitative study used reflexive thematic analysis and a framework approach [19] to explore experiences of CRF in people with BT. Approval was granted by The University of Sydney Human Research Ethics Committee (Project number: 2022/374).

### Participants

Three participant groups were recruited: (1) people with BT; (2) primary caregivers of people with BT; and, (3) healthcare professionals (HCPs) providing care for people with BT. Recruitment strategies included social media advertisements on twitter (X) circulated by the author team; study advertisements circulated via Australian professional organisations (i.e. Psycho-oncology Co-operative Research Group [PoCoG], Cooperative Trials Group for Neuro-Oncology [COGNO], and Clinical Oncology Society of Australia [COSA]) and not-for-profit organisations in Australia (i.e. Peace of Mind Foundation and Brain Tumour Alliance Australia [BTAA]) and presentations at BTAA patient education and information public fora. People with lived experience of primary BT (i.e., patients or caregivers) were eligible if they lived in Australia; HCPs if they provided care to adults (> 18 years old) with BT in Australia or New Zealand. This sample contributed to a parallel study conducted by the author team pursuing different research questions [20].

### Measures

Demographic and clinical/caregiving/professional characteristics of participants were collected using an online Qualtrics survey [21]. CRF experiences were explored using open-ended questions in a semi-structured interview format (Supplementary File 1). People with BT and caregivers were asked about their experience with CRF, support received, and how they managed fatigue. HCPs were asked about their approach to identifying and managing CRF in this population.

### Procedure

The online study invitation was distributed through relevant recruitment channels and included a link to the participant information sheet explaining the study, consent form, and demographics survey. Semi-structured interviews were conducted in English via online videoconference by members of the research team (RC, PhD, female; HB, BPsych Honours, female; or TC, BPsych Honours, Male). All interviewers were employed in full-time research positions, trained in qualitative research methodology, and had no prior relationship with interviewees. Only the interviewer and participant were present during interviews. All interviews were audio-recorded for transcription in Trint [22]. After reaching thematic saturation [23], three member-checking interviews were conducted with people with BT who had not previously been interviewed. During member-checking interviews, researchers assessed whether the thematic analysis accurately reflected participants’ experiences and/or opinions.

### Data analysis

Participant characteristics were analysed using descriptive statistics. Categorical data were reported as percentages and continuous data as medians and (inter-quartile) ranges.

Trint [22] audio transcription software was used to generate verbatim transcripts of interview audio-recordings. Once de-identified and checked for accuracy against original audio-recordings, three transcripts were selected at random for analysis. Members of the research team (RC, HB, TC, MF, MJ, HD, and JS) independently reviewed transcripts and applied preliminary “codes” to passages of potential relevance. Based on these transcripts, researchers collaboratively developed a coding framework of clearly described themes and subthemes. The coding framework evolved iteratively and was applied to subsequent transcripts using NVIVO [24]. The research team met regularly to review codes, discuss interpretations, resolve coding disagreements, and modify the coding structure.

Reporting adhered to the 32-item consolidated criteria for reporting qualitative research [25] (COREQ; Supplementary File 2).

## Results

### Demographic characteristics

Forty participants, with median age of 51 years (IQR = 14.8), completed the survey and an interview. Median interview duration was 41 min (IQR = 14).

Among people with BT (*n* = 24), there was relatively balanced representation across gender, tumour grade at diagnosis, and time since diagnosis. The majority (58%) were diagnosed with a glioma, four (16%) of which were glioblastoma multiforme (GBM). Most (46%) received a combination of treatments (surgery, radiation therapy, and chemotherapy) or surgery (21%), chemotherapy (8%), or radiation therapy (8%) alone. All caregivers (*n* = 5) were female, caring for their spouse.

Most participating HCPs (*n* = 11) were female (82%), working in an outpatient hospital setting (73%). HCPs represented a broad range of clinical specialties and years of experience working with people with BT. The typical patient profile cared for by HCPs was someone with GBM (91%) aged 50–65 years (73%).

See Tables 1, 2, and 3 for detailed participant characteristics.

**Table 1:** Sociodemographic and clinical characteristics of people with BT

Sociodemographic and clinical characteristics—people with BT	*N* (%)
Median age:	51 years (IQR = 16.0) ^a^
Gender	**24 (100)**
Male	10 (42)
Female	13 (54)
Not specified	1 (4)
Tumour type ^b^	**24 (100)**
Glioma	14 (58)
* Glioblastoma multiforme (GBM)*	*4 (16)*
Meningioma	4 (16)
Other ^c^	5 (21)
Not specified	2 (8)
Tumour grade at diagnosis	**24 (100)**
Grade I	3 (12)
Grade II–III	10 (42)
Grade IV	4 (17)
Not specified	7 (29)
Years since diagnosis	**24 (100)**
≤ 2	5 (21)
3 to 4	5 (21)
5 to 7	5 (21)
≥ 8	7 (29)
Not specified	2 (8)
Treatment received ^d^	**24 (100)**
Surgery	15 (63)
Chemotherapy	13 (54)
Radiotherapy	13 (54)
Not specified	3 (13)

This table is replicated in the report of a parallel study conducted by the author group with the same sample (20)

^a^Three participants did not provide this information

^b^One participant had received two separate diagnoses 6 years apart

^c^Includes oligoastrocytoma, atypical colloid cyst, mixed germ cell, and ganglioneuroblastoma

^d^Multiple answers possible

**Table 2:** Sociodemographic characteristics of caregivers

Sociodemographic characteristics—caregiver	*N* (%)
Median age:	52 years (IQR = 9.5) ^a^
Gender—female	**5 (100)**
Person they care(d) for—spouse	**5 (100)**
Patient’s tumour type	**5 (100)**
Glioma	3 (60)
Not specified	2 (40)
Patient’s tumour grade at diagnosis	**5 (100)**
Malignant (Grade III–IV)	2 (40)
Not specified	3 (60)
Length of care provided	**5 (100)**
≤ 2	2 (40)
3 to 4	1 (20)
5 to 7	0 (0)
≥ 8	1 (20)
Not specified	1 (20)

This table is replicated in the report of a parallel study conducted by the author group with the same sample (20)

^a^Two participants did not provide this information

**Table 3:** Sociodemographic characteristics of HCPs

Sociodemographic characteristics—HCP	*N* (%)
Median age:	43.5 years (IQR = 10.5) ^a^
Gender	**11 (100)**
Male	1 (9)
Female	9 (82)
Not specified	1 (9)
Primary clinical specialty	**11 (100)**
Oncology ^b^	4 (36)
Nursing	3 (27)
Occupational therapy	2 (18)
Palliative care	1 (9)
Not Specified	1 (9)
Healthcare setting ^c^	**11 (100)**
Hospital (inpatient)	2 (18)
Hospital (outpatient)	8 (73)
Other ^d^	2 (18)
Not specified	1 (9)
Years of experience working with BT patients	**11 (100)**
1 to 5	3 (27)
6 to 10	3 (27)
> 10	4 (36)
Not Specified	1 (9)
Approximate number of BT patients seen each year	**11 (100)**
< 50	5 (45)
50 to 100	2 (18)
> 100	3 (27)
Not specified	1 (9)
Most common tumour type seen	**11 (100)**
Glioblastoma multiforme (GBM)	10 (91)
Not specified	1 (9)
Typical age profile of BT patients seen	**11 (100)**
50 to 65	8 (73)
> 65	2 (18)
Not specified	1 (9)

This table is replicated in the report of a parallel study conducted by the author group with the same sample (20)

*HCP* healthcare professional, *BT* brain tumour

^a^One participant did not provide this information

^b^Includes medical oncology, neuro-oncology and radiation oncology

^c^Multiple answers possible

^d^Includes community organisations and in-home rehabilitation

### Qualitative findings

Four main themes were identified, encompassing 13 subthemes. The main themes were as follows: pervasiveness of fatigue; impact of fatigue; advice and support; and self-management strategies. Illustrative quotes are provided for each theme and subtheme with unique participant identifiers (coded as P = people with BT, C = Caregiver, H = Healthcare Professional). See Table 4 for additional illustrative quotes pertaining to each theme/subtheme.

**Table 4:** Illustrative quotes for themes and subthemes

Theme	Subtheme	Attribute	Quote
Theme 1: Pervasiveness of fatigue	*1.1. Universality of fatigue*	Universality	“It’s something that comes up with every patient in every single consultation” [H4] “I think it’s one of the consistent symptoms across the cohort that I see…around the 80% that just fatigue is one of the severe symptoms.” [H16] “It probably is one of the more significant problems for us.” [C3]
		Individual differences	“It varies from person to person… it depends on where the tumour is and that sort of thing” [H8] “I wouldn't say it’s been an overwhelming problem for me…But, you know, life can sometimes be tiring” [P31] “Just being alive was exhausting.” [P7]
		Uniqueness of CRF in people with BT (e.g. ABI-related factors)	“It's very different to a general cancer-related fatigue.” [H13] “That’s why [fatigue] pretty much affects everybody… because of what we’ve done to them and the fact that the tumour is still there interrupting connections.” [H12] “Because they took the tumours out of my left frontal cortex, I’ve got one channel open. So that produces its own kind of fatigue.” [P3]
	*1.2. Manifestation of fatigue*	Physical	“Every day he has 3 h sleep…so our days revolve around his sleep.” [C5] “There were days when I could barely walk around, I was just exhausted” [P13] “I had one event where I fell asleep at the traffic lights. You know, just nodded off. I still had one foot on the brakes so I didn't go anywhere and got woken up by the cars blowing horns at me.” [P17] “It’s just I’ve some days I’ve pretty much slept for 12 h and then I just sleep at night. I got no, and it’s not just energy, it’s strength.” [P30]
		Emotional	“I think it manifests most commonly in people’s level of agitation or becoming very overwhelmed cognitively…Fatigue doesn’t come up in [yawn] “I’m tired”. It comes up in [agitated noise], you know, people get a bit agitated or a short fuse or that sort of stuff.” [H12] “I definitely have learned when I’m getting too tired, I get a little bit teary” [P13] “I think there’s a significant element of psychological fatigue as well.” [H3]
		Cognitive	“Sometimes I can hear what I want to say in my head, but I can’t quite get it out of my mouth if I’m too tired.” [P13] “Everything is slower. So by the time they’re even at the door, they’re exhausted; because it’s hard to think and it takes so much more out of them.” [H16]
	*1.3. Pattern over time*	Persistence	“I didn’t feel normal again for about five years, I reckon. Well, my new normal at least.” [P45] “It’s not as bad as what it was, but it’s still there, you know.” [P33] “I still feel tired. I still have the after-effects of tiredness” [P16]
		Changes over time	“It is a progressive disease and it can be a progressive decline. You know, often the sort of last six, well 3 to 6 months of life, you know, they may well experience worsening fatigue.” [H3] “It has varied over time and depending on where I was at in the whole roller coaster of brain tumour. Pre-surgery, I was tired all the time. After surgery… I was sleeping something like 22 h a day. And then I, you know, I improved over time and then started going backwards again.”[P7] “It would be like one week, I can do a lot of efforts and not too tired and in another week having a shower is already exhausting. So there is no way to predict anything… it’s very hard to manage.” [P12]
		Awareness and expectations about fatigue	“I'm pretty sure that they warned me much more than what I experienced… maybe it’s a good thing that my expectations were worse than what I actually was.” [P13] “The treatment makes me more fatigued. It was expected, but I didn’t realise I would be so tired that I will have to stop work.” [P12] “I think I underestimated how long it lasts you know, fatigue.” [P8] “People have an enormous amount of hope that their lives, even though these big things happened, that their lives are still going to go back to what they were like.” [H12]
Theme 2: Impact of fatigue	*2.1. Worsened BT symptoms*	General	“[For] anyone who has a neurological problem, that problem is worse when they’re fatigued.” [H13] “It [fatigue] kind of jumbles your brain… my brain’s already a bit sizzled, it doesn’t need any further complications. So fatigue, um, really does make it worse.” [P1] “It can come out as increased seizures if people don't manage their fatigue correctly” [H12]
	*2.2. Psychosocial impacts*	Psychological impacts	“I was mentally and physically ruined. Absolutely ruined.” [P39] “They’re so tired and whacked out…and the motivation sometimes just isn’t there.” [H4] “I think depression follows quite closely with, of course, having your brain completely mucked up.” [C2]
		Social/relational impacts	“I find that it’s, you know, when I’m going to bed early…home life suffers.” [P38] “I had a two year old who yeah, I didn’t really have the capacity to engage for lengthy periods with during that period” [P33] “It had impacted also my relationship with people in general. Because if I’m tired, I get more irritable. Less patient.” [P12] “The wider social group often shrinks, because they’re too tired to engage.” [H17]
	*2.3. Functional impacts*	Work/study impacts	“I've been really rocked by not only fatigue, but mental lethargy and brain fog…to the point that it’s actually stopped me from working.” [P5] “I fell asleep at my desk a few times at work.” [P17] “Sometimes, like when I’m having a lecture or, you know, studying for a test, it especially affects me because I, I just lose my focus and literally can’t do anything.” [P21]
		Impact on everyday tasks	“You can’t, you just can’t live a normal life. Our lives are put on hold.” [C2] “I’ve had days where I’ve just stayed in bed and only got up to eat and drink.” [P45] “Down the track you know, we’re talking about “do they have the energy to have a shower? Do they have the energy to walk to the mailbox?” Those sorts of things.” [H16] “Everything is exhausting when you've got brain cancer” [C2]
		Impact on ability to engage in enjoyable activities	“I used to be a very keen reader and I feel like I’ll start a book, I’ll like it, I'll want to keep reading it, but I can’t be bothered.” [P31] “I don’t have the energy to drive, you know, the 20 min to the beach and try and find a park” [P42] “It would be nice to sort of have a holiday and maybe travel somewhere…But because of my level of fatigue, I’m not sure if I’m capable of driving my car for long distances or…actually have some energy to enjoy where you are and what you’re doing.” [P46]
Theme 3: Advice and support	*3.1. Fatigue assessment*	Informal assessment	“I sort of go by just observing and assessment within the home… because it’s just it’s not whether they’ve got fatigue – they’ve got fatigue. It’s pinpointing what level.” [H16] “It’s mainly a verbal thing. And I think that’s a problem… you really rely on that point of care conversation” [H3] “Very occasionally I use the FACIT-fatigue.” [H12]
		Lack of assessment	“There wasn’t really any, any real focus on it [fatigue], as part of conversations with the oncologist, [P38]
	*3.2. Advice from HCPs*	Pharmacological	“You do resort to medication—temazepam, you know, melatonin” [H4] “So first I will look for underlying causes that are reversible. So thinking about anaemia, medication, inappropriate medications and thyroid function.” [H17]
		Non-pharmacological	“I very much stress the value of regular exercise because we know from research that exercise actually helps moderate those effects of fatigue.” [H7] “I always recommend to everybody 100% of the time who has brain tumour, no matter how high functioning they are, to have a daily rest” [H13] “Lifestyle management. So eating well, getting enough sleep, drinking enough water and getting some exercise” [H12]
		Perceived helpfulness	“Once they got the level of it [medication] right or what was right for me I was able to function okay.” [P45] “I got told that I was running on empty. And I’m like, [chuckling] I could tell you that.” [P4] “The physio was saying I should never use more than 75% of my energy. To make sure I always have a bit of energy left for the day. And I try but I never know how much I have”. [P12]
		Lack of advice and support	“I asked them about [fatigue], but all they could say is that it will get better. You know, it’s a common side effect. And that’s it.” [P21] “I went to my GP and just said, “Look, I just feel exhausted…Mentally, physically, you know, just fatigued”. And I wasn’t really referred to anybody” [P38] “I pretty much received little to no support after I was having my treatments and things like that.” [P15]
	*3.3. Recommended information resources*	Recommended resources	“We have a single A4 page on strategies to manage fatigue…pacing, regular exercise etcetera. How many patients actually read it? That’s a whole other question.” [H7] “We print eviQ patient information and it does talk about fatigue, but on a very basic level.” [H11] “I sometimes give out a sleep handout, if that’s a contributor. Like a sleep hygiene hand out.” [H17]
		Lack of resources	“I do find that a lot of the resources are fairly vague.” [H3] “There was no sort of suggestion that here is a particular resource or a particular path that you can take to combat fatigue.” [P2] “But there’s no fatigue brochures. Like, you might open a brochure and there might be like a paragraph on fatigue.” [C5]
		Lack of CALD resources	“Doing things in languages other than English is definitely on our target list, but we just haven’t had the time nor the resources to that very well.” [H12] “The only book that I know that is in other languages is the BTAA^a^ booklet” [H4]
	*3.4. Supportive environments*	Family and friends	“If I have to leave early, no one complains. If I say, okay, that’s enough for me, I have to go home now. You know, everybody’s very understanding.” [P29] “If I’m well rested and pleasant, then it’s easy to create an environment for him to be well-rested and calm” [C4] “I think a lot of it’s about your mental toughness as well, whether you’re prepared to push through that and do you have people around you that encourage you.” [P10] “People meaning to support you, I think, also is fatiguing… it’s tiring because they impose their emotions or their worries or stresses” [P8]
		Work	“I haven’t been put under any pressure to do the things I can’t do.” [P46] “I was pretty lucky. I had a very supportive manager who did everything he could to support me back into the workplace. I don’t think I would have made it back if it wasn’t for him because he was just so supportive” [P33] “I fell asleep at my desk a few times at work. But my boss knew that I was getting over the radiation” [P17]
		Patient advocacy and support groups	“I’m part of another group, one about brain injury. And their symptoms and difficulties are all the same, So I relate very well with them and the way they manage their own fatigue. And yeah, having exchange about our experience is very good.” [P12] “The good part with [support groups] is you relate to them because you know exactly what it’s like…But the other side of it is we’re all very different.” [P31] “Having someone like [Peace of Mind Foundation] on your side is really good” [P24]
Theme 4: Self-management strategies		Lack of support → need to self-manage fatigue	“The bottom-line stuff, which is that the day to day stuff is not their [HCPs] realm. So we do have to sort a lot of stuff out ourselves.” [C2] “The patient does have to manage a lot of different causes of fatigue, um, but then also actually just the impacts of it.” [P1] “I just tried a whole lot of things” [P29]
	*4.1. Lifestyle*	General	“The exercise, sleeping and eating produced amazing energy levels.” [P39]
		Exercise	“I force myself to walk every day. And I think that helps… And it’s kind of counterintuitive because the last thing you want to do is exercise.” [P8] “When [fatigue] really hits me, I have to get out and go for a walk down the street in the city here just to get my brain back” [P33]
		Diet	“If I eat just crap, I feel really bad and it's like a hangover or like a really bad jetlag…if your body is not right, then it makes you more tired and it’s a vicious cycle.” [P8] “Lots of fluids…whether it be water or juice or something. But a lot of water seemed to always help.” [P30]
		Sleep	“I really think consistency in your sleep patterns is really important.” [P1] “Pretty much every day I’ll just go have a lie down and have a bit of a snooze, probably for about 20 min. And I find that helps.” [P16]
		Mindfulness	“The main thing I find kind of useful is just practising a bit of mindfulness…just let my brain rest for a few minutes.” [P21] “Taking one day at a time is important. Like being in the moment, being here now.” [P3]
	*4.2. Accepting new normal*	Accepting limitations	“I don’t keep up with the chores like I used to. I still manage to get things done, but yeah, I’ve learnt to let go.” [P15] “Sitting on the couch with a book on a Saturday is okay. You know, rather than having to feel like I achieve a lot of things.” [P13] “I guess I’ve just learnt that it’s not worth it pushing through, if I’m really tired. I have to kind of just take some time out” [P31]
		Balancing energy, having a routine and planning ahead	“Be open to a routine. So when is it best for [patient] to have a rest so that [they] can be better at a certain time?” [C2] “You have to really say, “Well, what does my week look like? What’s important and what’s not? And what can move? What can go?” [P1] “I had to teach myself how to say no to things” [P13]
		Being intentional with how they use their energy	“Having some sort of major purpose has helped reenergize me.” [P2] “It's about keeping the focus off myself…it was more about shifting the focus off my circumstances onto what are the things that are meaningful to you.” [P10]
	*4.3. Seeking social and informational supports*	Asking for help and communicating needs	“I have explained, and they have understood, [the effects of CRF] to my family. And some of my friends. And they get it.” [P29] “I’ve spent some time trying to educate [wife], just trying to help her understand that every step I take is an effort.” [P3]
		Information seeking	“Things that I research identify things like exercise and mindfulness as sort of key components.” [P2] “I’ll sit on the Internet and research all night on all this type of stuff.” [P33]

^a^BTAA = Brain Tumour Alliance Australia

### Theme 1—Pervasiveness of fatigue

#### Universality of fatigue

Overall, CRF was described as “such a different type of tiredness” [P13] infiltrating every area of one’s life:

I feel like I’m withering away... And I might not look exhausted, but I feel exhausted [P4]

Some HCPs noted fatigue in people with BT presents unique challenges compared to other cancers. As BTs are a form of acquired brain injury (ABI), it manifests similarly to ABI fatigue:

They’re people who’ve got brain injuries. And fatigue related to a brain injury I think trumps cancer-related fatigue [H13]

The extent of CRF varied among participants; however, all people with BT reported experiencing fatigue at some point after diagnosis. All HCPs stated CRF was a prevalent issue for their patients, describing it as an “almost completely universal symptom in brain cancer” [H1]:

Fatigue is a huge issue. It’s one of the most difficult symptoms, most bothersome symptoms for patients, and the most difficult to resolve. [H17]

#### Manifestation of fatigue

CRF was described as manifesting physically, emotionally, and cognitively. Participants most commonly discussed the physical experience of fatigue, typically characterised by persistent feelings of exhaustion unalleviated by sleep:

I was sleeping 16 hours a day... I just didn’t have the energy to walk more than 200 metres [P24]

I couldn’t sleep enough to stop the fatigue. [P39]

The cognitive experience of fatigue was broadly described as a “clouded, foggy mind” [P39], which included memory, concentration, and language issues, and becoming easily over-stimulated:

I think the brain gets very overwhelmed... The brain has to work very hard with all the stuff that we’ve done to it... It’s just having trouble coping. [H12]

Emotional aspects of fatigue involved being easily overwhelmed, agitated, and having difficulties regulating emotions. Some people with BT also noted the psychological toll of processing the terminal nature of their disease and its impact on their fatigue:

It shortens your temper [laughs]. So there is some sort of emotional regulation that does get impacted by fatigue. [P1]

I do think with fatigue there’s an emotional toll that makes you very tired right at the start because you’re negotiating... coming to terms with yourself. [P31]

#### Pattern over time

Treatment, medication changes, and disease progression contributed to variations in CRF both within and between participants. However, for most, fatigue was a pervasive side effect persisting long after completion of treatment:

I’ve still got that hangover of tiredness from the treatment. [P34]

Some participants expected to be fatigued, especially during treatment, or were warned about CRF by a HCP. However, many felt unprepared for and surprised by the persistence and severity of their fatigue post-treatment:

I don’t think I was prepared for the long-term fatigue. I was sort of prepared for it during that chemo and radiation period and maybe thought that it would get better [P13]

No one says fatigue is an ongoing symptom [C5]

### Theme 2—Impact of fatigue

#### Worsened BT symptoms

Patients frequently described an “overwhelming sense of fatigue” [P45] with major impacts on various aspects of life. For some people with BT, fatigue exacerbated pre-existing BT symptoms. For example, some participants reported fatigue compounded cognitive deficits resulting from the tumour location and/or treatment(s). Others reported fatigue was associated with increased seizure risk and neurological deficits (e.g. vestibular issues and hemispatial neglect):

[Fatigue] impairs their brain function, which means their symptoms are worse. [H13]

It increases my risk of seizures... in my case, fatigue might mean that I spend two days in hospital after having a seizure. [P1]

#### Psychosocial impacts

Participants commonly reported major impacts of fatigue on their relationships and mental wellbeing. Psychological impacts included low motivation, depressive symptoms, and reduced self-confidence:

My own self-confidence... in what I’m doing is really impacted because I often find that I lose track of where I’m up to in a conversation or in speech. [P1]

I think it’s also distressing because it [fatigue] is seen as an indicator that their cancer is not under control. [H17]

Several participants noted normal social interactions and group gatherings were tiring and effortful, “shrinking” their social groups. The constant desire for sleep had further detriment to personal relationships and home life, as patients lacked the energy to spend time with loved ones:

I think the thing that I struggle with the most is when my sister and her little kids come over from overseas and I want to spend so much time with them, but they just exhaust me. [P13]

Emotion regulation problems caused by CRF were reported to contribute to issues in friendships, familial, and intimate relationships. This relational tension was exacerbated when friends and family did not understand the extent of the patient’s fatigue:

[Fatigue’s] not accepted by a lot of people close to me. Like my soon-to-be very ex-husband... he just called me lazy and things like that. [P4]

Some participants also noted severe CRF is often associated with high caregiver burden, which in turn has consequences for the caregiver’s wellbeing:

It definitely affected us socially... Because of course, the carers, we have to do everything because they’re just too exhausted to do anything. [C2]

#### Functional impacts

Participants most frequently discussed the functional impacts of fatigue, namely their inability to work, perform everyday tasks, and engage in enjoyable activities. Most people with BT reported having to reduce or stop work/study due to the physical and cognitive impacts of their fatigue:

I still only work three and a half days a week because of the fatigue. [P13]

Likewise, “day-to-day stuff” such as grocery shopping, basic hygiene (e.g. showering), cooking, and cleaning were described as tiresome activities for people with BT.

I remember [patient] being too tired to eat... he would say, “I actually can’t be bothered eating” because he was just too tired. [C2]

The patient’s constant need for sleep, neurological deficits, and reduced motivation limited their ability to engage in fulfilling activities such as hobbies or travel:

Reading is something I don’t do anymore. Because, you know, five words – it just- I don’t – I can’t focus anymore because of the fatigue. [P42]

Your whole life is a routine… like you can’t really be spontaneous… [because] if they don’t sleep it’s just terrible. [C2]

### Theme 3—Advice and support

#### Fatigue assessment

Almost all HCPs reported routinely assessing patients’ fatigue through informal enquiry. Time constraints and workloads were mentioned as barriers to formal fatigue assessment:

We don’t use any measures. I’d like to, but we haven’t had that capacity at the moment... so it’s mainly a verbal thing. [H3]

In contrast, most people with BT and caregivers reported little to no discussion about or assessment of CRF. Some participants reported that even if these discussions happened, there was no follow-up support or advice offered:

They’d ask about energy levels... but there were never really any offers or suggestions on how to manage it. It was just, “Oh, well, that’s no good – moving on to the next thing”. [P7]

#### Advice from HCPs

All HCPs reported providing CRF support and advice to their patients. In contrast, very few people with BT or caregivers reported receiving help from their HCP in managing fatigue. Some people with BT attempted to raise the topic of fatigue with their HCPs and still did not receive the support they desired:

I went to my GP and just said, “Look, I just feel exhausted” ... And I wasn’t really referred to anybody [P38]

Several HCPs discussed the importance of addressing “underlying causes” [H17] that can be reversed through pharmacological intervention. Some participants experienced significant improvements in their fatigue once steroids and other medications were adjusted or introduced. However, most found pharmacological interventions ineffective in managing their fatigue:

You end up having to put them on things like melatonin just to get them into a decent sleep pattern. [H4]

Largely, the solution that I was given by my GP was just throw sleeping pills at it. [P7]

All HCPs reported providing non-pharmacological advice to their patients. This included physical activity, taking time off work, increasing sleep, establishing routines, and referral to allied health professionals (e.g. physiotherapists):

It’s all about that pacing, planning, and prioritising [H16]

Again, the perceived helpfulness of non-pharmacological support varied among participants:

[HCP] just signed me off work for like two weeks... I’m not sure it really helped at all, to be honest. [P38]

We discussed logging activity and breaking them down in small chunks to make sure I don’t use all my energy credits all at once. And yes, it helped a lot. [P12]

#### Recommended information resources

All participants identified a lack of CRF information resources specific to people with BT and their caregivers. HCPs said they recommended the Survivorship Diary [26], Building the Bridge [27], and Cancer Council [28, 29] resources. However, they conceded none was specific to CRF in people with BT:

It’s generally the Cancer Council stuff. It’s not anything specific about [BT-related] fatigue. And, you know, is there actually anything out there? [H5]

Patients and caregivers expressed frustration at the lack of resources covering the period beyond diagnosis and treatment, including long-term CRF:

I simply didn’t have that information at the end of my recovery period. I just had to sort of go and experience things and work it out from there. [P46]

No participants were aware of CRF resources available in languages other than English.

#### Supportive environments

Some people with BT discussed the role of family, friends, work environments, and formal support groups in navigating their fatigue. Given fatigue had a critical impact on employment, some people with BT stated workplace support (or lack thereof) had a notable impact on their ability to manage fatigue. A couple of people with BT had supportive employers who made their recovery easier by alleviating pressure and providing flexible work arrangements. While others were forced to reduce or stop work, as they were unable to maintain pre-diagnosis levels of productivity:

[Employer] said, you know, “if you can’t do it by the end of the financial year...we’ll make you redundant”. [P13]

Friends and family reportedly supported people with BT by providing compassion and/or accountability in areas such as exercise:

I joined a group which made me almost obligated to paddle so on those mornings when I was struggling to get out of bed, I’ve just got to; the guys will be there. [P10]

Few people joined formal support groups due to the lack of groups specific to BT and/or CRF. The perceived value of support groups varied among participants:

I went to a couple of group sessions and they kind of depressed me. [P13]

### Theme 4—Self-management strategies

Due to the lack of CRF advice and support provided, people with BT and caregivers felt the need to develop their own management strategies. Almost all people with BT reported going through a process of trial and error to develop strategies that may work for them.

#### Lifestyle

Most participants implemented general lifestyle changes to help manage their fatigue. This included increasing physical activity, “eating healthy”, monitoring caffeine levels, avoiding alcohol, napping, improving sleep hygiene, and practising mindfulness. Some also proactively scheduled in time to sleep during the day to alleviate fatigue.

Exercise, it’s quite good. I cleaned up my diet, I don’t drink...it’s really cliche, but it's just the normal stuff people tell you to do to take care of yourself. [P1]

I quickly learned the best way to deal with fatigue is just have a sleep. [P3]

#### Accepting new normal

Some participants said accepting their limitations, listening to their body, and having self-compassion were key in managing their fatigue. This enabled people with BT to plan activities with their capabilities in mind and effectively balance their energy levels:

It's very much about managing what I'm doing in any given day and allowing space to just do nothing and recover from whatever it is that I've been doing. [P7]

A lot of it for me personally, was about sort of giving myself permission to stop and to take breaks. [P7]

Some participants reported that due to a global lack of energy and motivation, they tried to intentionally “spend” their energy on fulfilling, enjoyable activities.

Part of managing fatigue is doing things you like doing. And that carries you through the fatigue. [P3]

#### Seeking social and information support

Some participants noted the importance of communicating their needs to family, friends, and HCPs to receive appropriate support. This included setting expectations for social events (e.g. needing to leave early) and requesting referrals to allied health professionals:

I was happy enough to sort of share with people rather than the old bloke thing of “keep it to yourself, you're supposed to be tough”... And I think that's why I could cope better. [P10]

The lack of information provided by HCPs led many people with BT and caregivers to seek information on their own. This included seeking information about the disease, causes of fatigue, and management strategies:

I went in, you know, quite oblivious to what I was in for and so... I did a lot of research. [P33]

**Fig. 1:**
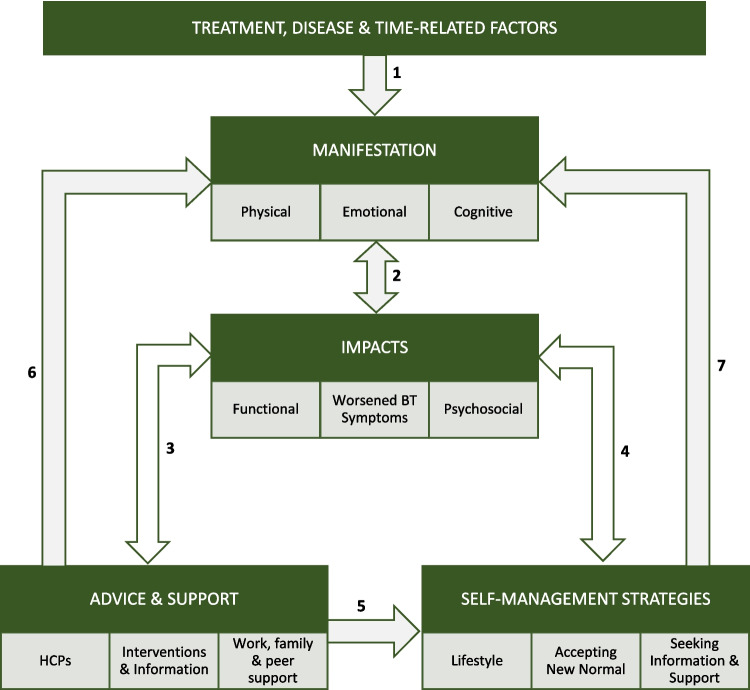
Model of CRF experienced in people with BT

See Fig. 1 for a visual representation of the experience and impact of CRF in people with BT based on our findings.

## Discussion

This study provides rich insights into the subjective experience of cancer-related fatigue and current management approaches from the perspective of people living with BT, their caregivers, and HCPs in Australia. Results identified the pervasiveness and diverse impacts of fatigue on people with BT, gaps in advice and support available, and the self-management strategies adopted by people with BT to manage their fatigue. Notably, both HCPs and people with BT reported a lack of targeted resources, emphasising a critical need for the development of evidenced-based supports for CRF tailored to people with BT.

Our findings emphasise the ubiquitous nature of CRF, with all participants reporting fatigue to be a substantial issue at some point throughout the disease and treatment trajectory. People with BT reported emotional, cognitive, and physical aspects of fatigue, aligning with the NCCN multidimensional conceptualisation of CRF [10]. This has important implications for assessing fatigue in this population as it suggests unidimensional measures capturing individual aspects of fatigue are not suitable for comprehensive assessment of CRF in research contexts. Rather, multidimensional measures such as the EORTC QLQ-FA12 [30], that generate separate scores for physical, emotional, and cognitive fatigue, will provide more holistic assessment of CRF. Clinicians should also probe into these different aspects when performing assessments of fatigue in clinical practice.

The longevity of fatigue post-treatment was reported to be particularly unexpected by both people with BT and caregivers, emphasising the need to routinely inform those affected by BT that fatigue may continue to persist long after treatment ends. This underscores the importance of conducting long-term follow-up assessment of CRF post-treatment with provision of appropriate support. To help HCPs overcome barriers to assessment identified in this study (i.e. time constraints and high workload), electronic systems could be implemented to assess fatigue using standardised questionnaires, with scores above a set threshold triggering alerts to the treating team for discussion with the patient, minimising clinician burden.

Similar to previous findings from studies examining CRF in people with lung [31] and other cancer types [32], participants reported impacts on diverse aspects of daily life. These included functional impacts such as inability to work, perform activities of daily living and engage in leisure activities such as hobbies or travel; inter-personal difficulties resulting from trouble communicating, lacking energy to socialise, and others not understanding the impact of CRF; and impacts on caregivers in terms of limiting their social life and needing to plan any activities far in advance. Impacts especially unique to people with BT included CRF exacerbating BT symptoms, particularly cognitive and neurological deficits and increasing risk of seizure. These potential impacts should be communicated to people with BT, their caregivers, family, and friends to facilitate understanding of CRF; help set realistic expectations about the potentially persistent and pervasive impact of fatigue on their functioning; and emphasise the importance of proactively managing CRF.

Results further indicated a stark discrepancy between patient/caregiver and healthcare professional experiences of CRF assessment and provision of support. Although almost all HCPs reported assessing fatigue by asking patients about fatigue during consultations and providing advice and support, both patients and caregivers reported experiencing little to no discussion of fatigue and a lack of any support. These findings align with previous research in general cancer populations indicating CRF is under-assessed and under-treated [33, 34]. The disparate reports between patients/caregivers and HCPs in this study may be because HCPs who participated in this study were more engaged and interested in CRF management and support. This means HCP awareness and management of CRF are likely less optimal in reality, as indicated by those affected by BT in our study. All participant groups reported a lack of CRF resources specific to people with BT and were not aware of any resources available in languages other than English, likely contributing to lack of provision of support from HCPs.

Due to the lack of support received, people with BT reported resorting to developing and trialling their own CRF management strategies to identify what works for them. The self-management strategies trialled by participants in our study included a range of lifestyle changes, accepting their new normal, and seeking social and information support. These findings align with results from qualitative studies in head and neck cancer survivors which similarly found patients self-manage symptoms through a process of trial and error, using a range of strategies, including adopting a healthy lifestyle (e.g. healthy diet, exercise, meditation), using support from others and accepting their illness and its consequences [35, 36]. Given existing evidence that self-management interventions can empower patients living with cancer to reduce their symptom burden [37, 38], and that people with BT are likely to have unique neurological and cognitive impairments that differ from other cancer types, future work should seek to identify effective self-management strategies in this population to inform the development of self-management interventions for CRF in people with BT.

Based on our findings, we developed a conceptual model (Fig. 1) to depict the experience of CRF in people with BT. The model begins with treatment, disease, and time-related factors which contribute (arrow 1) to the exact manifestation of fatigue. The way in which fatigue manifests, in turn, reciprocally impacts (arrow 2) on the person with BT’s functional ability, psychosocial interactions, and BT symptoms. To cope with these impacts, the person with BT will seek advice and support (arrow 3). The provision (or lack) of advice and support from HCPs, work, family, and peers, in turn, can either alleviate or exacerbate the impact and manifestation of fatigue (arrows 3 and 6). To further cope with CRF impacts (arrow 4), and in response to advice and support (or when it is lacking; arrow 5), people with BT often trial self-management strategies to manage CRF. The effectiveness (or lack thereof) of these management strategies can either mitigate or exacerbate the impact and manifestation of fatigue (arrows 4 and 7). This model can be used to create awareness among HCPs of how CRF manifests and impacts people with BT and the crucial role of HCP support in appropriately managing CRF.

Similar to findings internationally [39, 40], our results indicate a clear clinical pathway for managing CRF in people with BT is lacking in Australia. Clinical pathways provide standardised, evidence-based multidisciplinary management plans, identifying an appropriate sequence of clinical interventions, timeframes, milestones, and expected outcomes for patients [41]. A clinical pathway for CRF would reduce variation in the identification and management of CRF and improve the quality of life of those with BT [42, 43]. A clinical pathway for managing CRF should include clear guidance on routine screening and standardised assessment methods and which evidence-based resources and interventions to recommend depending on patient characteristics and fatigue severity. To facilitate this, existing interventions that have shown efficacy in alleviating CRF in other cancer populations [44] should be evaluated in people with BT. Although our findings indicate the manifestation and impact of CRF in people with BT is generally similar to other cancer types [31, 32], the unique neurological and cognitive deficits experienced by people with BT necessitate targeted evaluation of evidenced-based interventions in this population with appropriate tailoring.

This study has some limitations. Although we garnered perspectives from diverse people with lived and professional experience of BT, there was relatively limited participation of caregivers and HCPs. In addition, all participants lived in Australia and were English speaking, reducing the generalisability of these findings to other countries and cultures, particularly regarding the types of resources and support provided for CRF by HCPs. People with BT who took part were those physically able and with sufficient cognitive capacity to participate in a 1-h interview; those who opted not to participate may have been even more impacted by fatigue. Finally, HCPs who were more interested in CRF management may have self-selected into the study, meaning CRF assessment and management practices are likely worse in reality.

This study identified gaps in current CRF management practices with important clinical implications. HCPs should proactively inform people with BT, both before and after treatment, that they are likely to experience persistent CRF that will impact their ability to perform their usual activities. Brain tumour healthcare teams should implement long-term standardised assessment of CRF post-treatment to identify those struggling with fatigue and in need of support. This could be achieved by administering validated fatigue screening measures at regular intervals following treatment completion to identify those with fatigue needing follow-up. To ensure patients are better informed and regularly assessed and supported for fatigue, there is a need for future work to identify context-specific implementation strategies of CRF assessments and supports to address barriers experienced by HCPs in their practice. Lastly, there is a critical need for future research to develop and evaluate tailored evidence-based resources and interventions for CRF in people with BT, to identify those suitable for implementation within healthcare systems.

## Conclusions

CRF is a pervasive symptom experienced by almost all people with BT, with major impacts on quality of life. Despite the prevalence of CRF, patients and caregivers perceived it is not prioritised or discussed in clinical encounters. Information and interventions designed to alleviate CRF in this population are lacking. A clear clinical pathway to identify and manage CRF in people affected by BT is urgently required.

## Supplementary Information

Below is the link to the electronic supplementary material.

Supplementary file1 (DOCX 46 KB)

## Data Availability

The datasets generated during and analysed during the current study are available from the corresponding author on reasonable request.
