# Supplementary material for: “Such a different type of tiredness”: people with brain tumour, their caregivers’, and healthcare professionals’ qualitative perceptions of cancer-related fatigue
Source: J Cancer Surviv. 2024 Oct 15;20(3):839–57. doi: 10.1007/s11764-024-01691-3 (PMC13144180; doi:10.1007/s11764-024-01691-3)
Supplement: Supplementary file 1 — Supplementary file1 (DOCX 46 KB) [file 11764_2024_1691_MOESM1_ESM.docx]

**Supplementary Files: “Such a different type of tiredness”: people with brain tumour, their caregivers’, and healthcare professionals’ qualitative perceptions of cancer-related fatigue**

Table of Contents

[**Supplementary File 1** 2](#_Toc170376999)

[**INTERVIEW GUIDE – Health professionals** 2](#_Toc170377000)

[**INTERVIEW GUIDE – Consumer representatives (patients & caregivers)** 5](#_Toc170377001)

[**Supplementary File 2** 8](#_Toc170377002)

[**Consolidated criteria for reporting qualitative studies (COREQ): 32-item checklist** 8](#_Toc170377003)

# **Supplementary File 1**

## **INTERVIEW GUIDE – Health professionals**

Introduction to study

*Start Recording.*

[*Spoken by interviewer*]- “This study aims to understand approaches currently used to manage cancer-related fatigue in people with brain a tumour; whether RESTORE is an appropriate intervention for people with brain a tumour; and, modifications required to tailor suitability of RESTORE for people with brain a tumour.

I would like to make an audio-recording of this interview. This will help make sure that I catch everything you say. The audio-recording of our conversation will be typed out in full. When we do that we make sure there is nothing in the document that could identify you so for example, if you mention your name or the name of where you work, that information will be blanked out. You may stop the interview at any time and the information provided will not be included in the study. You may also refuse to answer any question that you don’t want to answer. Before we start, can you please confirm you are happy to continue?

*[If participant responds yes, continue]*

*[Spoken by interviewer]*- “Thank you for volunteering your time. Are you ready to begin?”

**Demographics and clinical experience** *[ completed before the interview using the online Qualtrics survey]*

1. Age
2. Gender
3. Primary clinical specialty
4. Healthcare setting
5. Number of years post specialist qualification (experience)?
6. Approximately how many people with brain tumour do you see in your practice each year?
7. What different types of brain tumour diagnoses do you see in your practice?
8. What is the most common brain tumour diagnosis seen in your patient population?
9. What is the typical age profile of the patient population that you see in your clinical practice?

**TOPIC ONE: Approach to CRF identification and management**

- In your experience, is cancer-related fatigue a significant issue for people with brain tumour?
  - How does it appear in these people?
  - How does it impact their daily activities?
- Do you routinely ask your patients about fatigue?
- [If yes] How do you typically assess and document fatigue (e.g. verbally during consultation, use a screening measure and if so what measure/instrument)?
- Are there any treatment/management strategies you recommend to your patients presenting with fatigue?
- [If yes] What determines which intervention is recommended to a particular patient?
- Do you give your patients any information materials to take home to read or direct them to any resources to help them manage their fatigue? If yes, which ones. If no, why not?
- Do you provide any resources/support for fatigue in languages other than English?

**TOPIC TWO: Feedback on RESTORE and required modifications**

*A video recording summarising the content of the RESTORE intervention and a link to the RESTORE webpage was sent to you before this interview.*

- Have you had time to review the video recording and any of the RESTORE modules?

*If the participant indicates* ***no****, provide an overview during the interview.*

- [if yes] Which of the five RESTORE modules did you review?
- Do you think the content is relevant and appropriate for people with brain tumour?
- Are there certain topics that should be emphasised more/less? Are any topics unnecessary?
- Are any important topics missing?
- Do you believe people living with a brain tumour would find RESTORE helpful? Why or why not?
  - In your experience, how do you think they would respond to RESTORE?
  - How capable do you think they would be to use self-management strategies?
  - Do you think caregivers would benefit from using RESTORE to support their loved one?
  - What factors/features would encourage patients to use RESTORE?
  - Are there other resources you would prefer to RESTORE?
- Do you think there are particular subgroups likely to benefit most/least from RESTORE?
- Do you think the format is appropriate for people with a brain tumour? (e.g. presentation, clarity, language, font etc.)
- What do you think is the best format for interventions for people with a brain tumour? (e.g. booklet, video or audio information, online intervention, in clinic, group-based, etc.)
  - Are there particular physical or cognitive changes in this population that would impact the optimal format?
  - Are there any other factors that might impact the optimal format?
- What other modifications to the content or format might be necessary?
- Are there any additional factors that might enhance the use of RESTORE?
  - Are there any factors that might act as a barrier and discourage use?
- If a modified version of RESOTRE for people with a brain tumour was available, would you recommend it to your patients?

Neutral probes will be used to elicit more information. For example:

- “*Can you tell me more about that?”*
- *“Can you think of anything else?”*

General follow-up probes will be used to elicit more information. For example:

- *“In what way?”*
- *“How do you mean?”*
- *“Can you give me an example?*
- *“Any other reasons?”*
- *“What makes you think this?”*
- *“What makes you feel that way?*"

## **INTERVIEW GUIDE – Consumer representatives (patients & caregivers)**

**Introduction to study**

*Start Recording.*

[*Spoken by interviewer*]- “This study aims to understand how people affected by brain tumour manage cancer-related fatigue; whether RESTORE is an appropriate intervention for people with a brain tumour; and, modifications required to tailor suitability of RESTORE for this group of people.

I would like to make an audio-recording of this interview. This will help make sure that I catch everything you say. The audio-recording of our conversation will be typed out in full. When we do that we make sure there is nothing in the document that could identify you so for example, if you mention your name or the name of where you live, that information will be blanked out. You may stop the interview at any time and the information provided will not be included in the study. You may also refuse to answer any question that you don’t want to answer. Before we start, can I confirm you are happy to continue?”

*[If participant responds yes, continue]*

*[Spoken by interviewer]*- “Thank you for volunteering your time. Are you ready to begin?”

**Demographics and clinical characteristics** *[to be completed before the interview using the online Qualtrics survey]*

1. Age

2. Gender

3. Have you previously been diagnosed with a brain tumour? Y/N

[If yes respondent proceeds to Q4-6; if no respondent proceeds to Q7-9]
4. What was your primary brain tumour diagnosis?

5. When did you receive this diagnosis?

□ 2 years or less □ 3-4 years □ 5-7 years □ 8 years or more

6. What treatments have you had?
 □ Surgery □ Radiation therapy □ Chemotherapy □ Other, please specify ………….

**Only answered by those who responded no to Q3* *

7. Have you cared for or provided support to someone living with a brain tumour? Y/N

8. What was this person’s primary brain tumour diagnosis?
9. For how long did you provide care or support to this person?

□ 2 years or less □ 3-4 years □ 5-7 years □ 8 years or more

**TOPIC ONE: Experience of CRF and its management**

- In your experience, is cancer-related fatigue a problem for people with a brain tumour?
  - What sort of impact does it have?
  - How does it affect daily life?

*If participant is a person with a brain tumour:*

- - Did you get any help to deal with your fatigue?
  - What type of help did you receive?
  - Who did you receive this help from?
  - Are there any helpful strategies you would recommend to others experiencing fatigue?

*If participant is a caregiver:*

- - Did you receive any help in supporting the person you were caring for with their fatigue?
  - What type of help did you receive?
  - Who did you receive this help from?
  - Are there any helpful strategies you would recommend to others trying to support someone struggling with cancer-related fatigue?

**TOPIC TWO: Feedback on RESTORE and required modifications**

*A video recording summarising the content of the RESTORE intervention and a link to the RESTORE webpage was sent to you before this interview.*

- Have you had time to review the video recording and any of the RESTORE modules?

*If the participant indicates* ***no****, provide an overview during the interview.*

- [if yes] Which of the five RESTORE modules did you review?
- Do you think the content is relevant for people affected by a brain tumour?
- Are there certain topics that should be focused on more/less? Are any topics unnecessary?
- Are any important topics missing?
- Do you think people affected by a brain tumour would find RESTORE helpful? Why or why not?
  - Do you think the self-management strategies are useful? Why or why not?
  - Do you think caregivers would benefit from using RESTORE to support their loved one?
  - Are there other resources you would prefer to RESTORE?
- What do you think of the format? (e.g. presentation, clarity, language, font etc.)
- What do you think is the best way of delivering support like this for fatigue? (e.g. booklet, video or audio information, online intervention, in clinic, group-based, etc.)
- Are there any other ways you think RESTORE should be changed for it to be more useful for people with a brain tumour ?
- Are there any other things you can think of that might improve the use of RESTORE?
- If an adapted version of RESTORE was created for people with a brain tumour, would you use it?

Neutral probes will be used to elicit more information. For example:

- “*Can you tell me more about that?”*
- *“Can you think of anything else?”*

General follow-up probes will be used to elicit more information. For example:

- *“In what way?”*
- *“How do you mean?”*
- *“Can you give me an example?*
- *“Any other reasons?”*
- *“What makes you think this?”*
- *“What makes you feel that way?*"

# **Supplementary File 2**

## **Consolidated criteria for reporting qualitative studies (COREQ): 32-item checklist**

| **No. Item** | **Guide questions/description** | **Reported on Page #** |
| --- | --- | --- |
| **Domain 1: Research team and reﬂexivity** |  |  |
| *Personal Characteristics* |  |  |
| 1. Inter viewer/facilitator | Which author/s conducted the interview or focus group? | 5 |
| 2. Credentials | What were the researcher’s credentials? E.g. PhD, MD | 5 |
| 3. Occupation | What was their occupation at the time of the study? | 5 |
| 4. Gender | Was the researcher male or female? | 5 |
| 5. Experience and training | What experience or training did the researcher have? | 5 |
| *Relationship with participants* |  |  |
| 6. Relationship established | Was a relationship established prior to study commencement? | 5 |
| 7. Participant knowledge of the interviewer | What did the participants know about the researcher? e.g. personal goals, reasons for doing the research | 5 – outlined in participant information sheet explaining study purpose |
| 8. Interviewer characteristics | What characteristics were reported about the inter viewer/facilitator? e.g. Bias, assumptions, reasons and interests in the research topic |  |

| **Domain 2: study design** |  |  |
| --- | --- | --- |
| *Theoretical framework* |  |  |
| 9. Methodological orientation and Theory | What methodological orientation was stated to underpin the study? e.g. grounded theory, discourse analysis, ethnography, phenomenology, content analysis | 5 |
| *Participant selection* |  |  |
| 10. Sampling | How were participants selected? e.g. purposive, convenience, consecutive, snowball | 5 |
| 11. Method of approach | How were participants approached? e.g. face-to-face, telephone, mail, email | 5 |
| 12. Sample size | How many participants were in the study? | 6 |
| 13. Non-participation | How many people refused to participate or dropped out? Reasons? | NA |
| *Setting* |  |  |
| 14. Setting of data collection | Where was the data collected? e.g. home, clinic, workplace | 5 |
| 15. Presence of non-participants | Was anyone else present besides the participants and researchers? | 5 |
| 16. Description of sample | What are the important characteristics of the sample? e.g. demographic data, date | 6-10 |
| *Data collection* |  |  |
| 17. Interview guide | Were questions, prompts, guides provided by the authors? Was it pilot tested? | 5 |
| 18. Repeat interviews | Were repeat interviews carried out? If yes, how many? | NA |
| 19. Audio/visual recording | Did the research use audio or visual recording to collect the data? | 5 |
| 20. Field notes | Were ﬁeld notes made during and/or after the interview or focus group? | NA |
| 21. Duration | What was the duration of the interviews or focus group? | 6 |
| 22. Data saturation | Was data saturation discussed? | 5-6 |
| 23. Transcripts returned | Were transcripts returned to participants for comment and/or correction? | NA |
| **Domain 3: analysis and ﬁndings** |  |  |
| *Data analysis* |  |  |
| 24. Number of data coders | How many data coders coded the data? | 6 |
| 25. Description of the coding tree | Did authors provide a description of the coding tree? | NA |
| 26. Derivation of themes | Were themes identiﬁed in advance or derived from the data? | 6 |
| 27. Software | What software, if applicable, was used to manage the data? | 6 |
| 28. Participant checking | Did participants provide feedback on the ﬁndings? | 6 |
| *Reporting* |  |  |
| 29. Quotations presented | Were participant quotations presented to illustrate the themes/ﬁndings? Was each quotation identiﬁed? e.g. participant number | 12-27 |
| 30. Data and ﬁndings consistent | Was there consistency between the data presented and the ﬁndings? | 12-27 |
| 31. Clarity of major themes | Were major themes clearly presented in the ﬁndings? | 12-27 |
| 32. Clarity of minor themes | Is there a description of diverse cases or discussion of minor themes? | 12-27 |
